# Bacterial microbiome of the chigger mite *Leptotrombidium imphalum* varies by life stage and infection with the scrub typhus pathogen *Orientia tsutsugamushi*

**DOI:** 10.1371/journal.pone.0208327

**Published:** 2018-12-06

**Authors:** Loganathan Ponnusamy, Alexandra C. Willcox, R. Michael Roe, Silas A. Davidson, Piyada Linsuwanon, Anthony L. Schuster, Allen L. Richards, Steven R. Meshnick, Charles S. Apperson

**Affiliations:** 1 Department of Entomology and Plant Pathology, North Carolina State University, Raleigh, North Carolina, United States of America; 2 Comparative Medicine Institute, North Carolina State University, Raleigh, North Carolina, United States of America; 3 Department of Epidemiology, Gillings School of Global Public Health, University of North Carolina, Chapel Hill, North Carolina, United States of America; 4 Department of Entomology, US Army Medical Component, Armed Forces Research Institute of Medical Sciences, Bangkok, Thailand; 5 Naval Medical Research Center, Viral and Rickettsial Diseases Department, Silver Spring, Maryland, United States of America; University of Reading, UNITED KINGDOM

## Abstract

Scrub typhus is a mites-borne rickettsiosis caused by the obligate intracellular Gram-negative bacterium *Orientia tsutsugamushi*. The disease is potentially life threatening and is prevalent in tropical Asia, islands of the western Pacific Ocean and northern Australia where an estimated one million cases occur annually. *Orientia tsutsugamushi* is transmitted by the bite of larval mites in the genus *Leptotrombidium*. In the present study, the composition of the microbiome in larvae, deutonymphs and adult males and females from laboratory colonies of *L*. *imphalum* that were infected as well as uninfected with *O*. *tsutsugamushi* were investigated by high-throughput sequencing of the bacterial 16S rRNA gene. Notably, the bacterial microbiomes of infected adult females were dominated by sequences of *O*. *tsutsugamushi* and an unidentified species of Amoebophilaceae, which together comprised 98.2% of bacterial sequences. To improve the taxonomic resolution of the Amoebophilaceae OTU a nearly full length sequence of the 16S rRNA gene was amplified, cloned, and Sanger sequenced. Infected female mites had 89 to 92% nucleotide identity with the Amoebophilaceae family, indicating that the bacterium was likely to be a species of a novel genus. The species composition of bacterial communities varied between mite life stages regardless of their infection status. Uninfected adults exhibited greater species diversity than adults infected with *O*. *tsutsugamushi*. In the infected colony, the rate of filial infection with *Orientia* was less than 100%. Larval and male mites that were PCR-negative for *Orientia* contained low numbers of sequences of Amoebophilaceae (0.01 and 0.06%, respectively) in their taxonomic profiles, suggesting that a mutualistic relationship exists between the novel species of Amoebophilaceae and *O*. *tsutsugamushi*. Our study findings provide the basis for further research to determine the influence of the novel Amoebophilaceae species on the bacterial microbiome and on vector susceptibility to and transovarial transmission of *O*. *tsutsugamushi*.

## Introduction

Scrub typhus, or tsutsugamushi disease, is a mite-transmitted rickettsiosis that occurs in much of Asia, northern Australia, and among islands in the western Pacific. Epidemiological data clearly demonstrates that there has been a rapid increase in the number of scrub typhus cases in recent years [1]. The disease is a significant source of morbidity and mortality, with an estimated one billion people at risk and approximately one million cases reported annually [2, 3]. The mortality rate of scrub typhus is variable (0%-70%) but may reach high levels in untreated patients with a median mortality rate of 6% [4]. Scrub typhus is caused by an obligate intracellular, Gram-negative bacterium, *Orientia tsutsugamushi* (formerly *Rickettsia tsutsugamushi*), placed in the genus *Orientia* within the family Rickettsiaceae [5, 6]. Larval *Leptotrombidium* mites, commonly referred to as “chiggers”, typically parasitize wild rodents and can transmit *O*. *tsutsugamushi* to humans via an infectious bite. The processes of cellular infection by *O*. *tsutsugamushi* have been nicely reviewed [7]. In contrast, the deutonymph and adult stages in the life cycle of *Leptotrombidium* mites are not parasitic and feed on detritus, insect eggs and soft-bodied invertebrates [8]. Although it is not known whether any exogenously acquired microbes are transstadially and/or transovarially transmitted in *Leptotrombidium* species, microbial endosymbionts are known to be vertically transmitted in their arthropods hosts [9–11].

Endosymbionts generally affect host biology and population dynamics by improving host arthropod fitness [11, 12]. A growing body of literature supports the observation that the microbiome of arthropods contains bacteria that act as facultative mutualists, providing protection from pathogen infection by activating immune responses [13–17] or through interference with transovarial transmission of pathogenic bacteria [14, 18]. In contrast, endosymbiotic microbes have also been noted to facilitate the transmission of pathogens by arthropods [19]. While the prevalence and transovarial transmission of *O*. *tsutsugamushi* in *Leptotrombidium* species has been previously investigated [20–24], the structure of the microbiome and bacterial community dynamics both with and without *O*. *tsutsugamushi* co-infection in mite vectors of scrub typhus has not been previously studied.

It has recently been shown that some symbionts, including species of *Cardinium*, *Rickettsia*, *Wolbachia*, and others, have evolved the ability to cause reproductive alterations in their arthropod hosts, such as feminization, cytoplasmic incompatibility, male killing and parthenogenesis [25–28]. The reproductive alterations effectively increase the frequencies of infected females in the host populations, often at the expense of host fitness. Effects of *O*. *tsutsugamushi* on *Leptotrombidium* mite biology appears to vary between species. *Leptotrombidium imphalum* and *L*. *chiangraiensis* mites infected with *O*. *tsutsugamushi* manifested significantly longer development times and decreased fecundity compared to mites that are not infected [29]. However, an earlier study [30] found no difference between infected and uninfected *L*. *deliense* mites in development time or fecundity. Collectively, earlier published studies [25, 26, 28] suggested or demonstrated that reproductive alterations in their arthropod host may be linked to the suppression of key members of the microbial community. Bacterial characterizations are needed for a better understanding of the role of bacteria in disease progression that can lead to novel strategies to manipulate arthropod-associated microbial communities to prevent disease. Furthermore, little is known about compositional differences in bacterial microbiome structure between life stages. In our present study, we compared the bacterial microbiome structure of infected and uninfected *Leptotrombidium imphalum* colonies. Specifically, for mites from these two laboratory colonies (that were maintained in identical environmental conditions), we compared the relative abundance of bacterial species in larvae, deutonymphs and adult males and females.

## Materials and methods

### Mite samples

*Orientia tsutsugamushi*-infected and uninfected *Leptotrombidium imphalum* larvae, deutonymphs, adult males and females, and eggs of *Sinella curviseta* (Collembola: Entomobryidae) used as a food source for the mites [23, 24, 31] were obtained from laboratory colonies maintained by the Armed Forces Research Institute of Medical Sciences (AFRIMS), Bangkok, Thailand. Colonies of *O*. *tsutsugamushi*-infected *L*. *imphalum* mites were established by collecting larvae infesting wild-rodents in Thailand. The Collembolan was originally obtained from the U.S. Army Medical Research Unit in Malaysia in 1993 and has been in continuous colonization in AFRIMS since that date. The colonies of infected and uninfected *L*. *imphalum* mites were maintained in an insectary in separate containers and arthropod containment rooms but under the same conditions of temperature, relative humidity and light regimens, and larval mites were fed on the same species of rodents [32]. The samples of mite life stages from the infected and uninfected colonies were preserved separately in ethanol and shipped to the NCSU for processing and DNA analyses.

### DNA extraction from *L*. *imphalum*

One hundred eighty-nine mite samples (114 infected and 75 uninfected) and 24 Collembola eggs (three eggs/pool) were prepared and DNA extracted. Mites and eggs were removed from ethanol and washed with sterile phosphate-buffered saline (PBS) five times. Genomic DNA was extracted from individual *O*. *tsutsugamushi*-infected and uninfected mites using a modified tissue protocol from the QIAamp DNA Mini Kit (Qiagen, Hilden, Germany). Each mite or egg sample was placed in a 2-mL microcentrifuge tube, and 180 μL of ATL lysis buffer and glass beads were added followed by pulse-vortexing to homogenize the sample. Ten microliters of Proteinase K solution (20 mg/mL) was added, and the sample was incubated at 56°C for 1 h. Subsequently, 200 μL of AL buffer was added, and the sample mixed by pulse-vortexing for 15 s followed by incubation at 56°C for 30 min. Two hundred microliters of absolute ethanol was added, and the sample mixed by pulse-vortexing for 15 s. The sample was then applied to a QIAamp spin column, and DNA was eluted in 50 μL molecular grade water. DNA quality and quantity were assessed (260/280 and 260/230 ratios) using NanoDrop 1000 Spectrophotometer (Thermo Fisher Scientific, Waltham, MA USA). The total genomic DNA of each mite and Collembola egg pool was normalized to a concentration of 10 ng/ μL and stored at −20°C until amplification.

### Detection of *Orientia tsutsugamushi* in mites

Conventional PCR was performed to verify the presence of *O*. *tsutsugamushi* DNA in individual mites (deutonymphs, larvae, adult females and males) from the infected colony of *L*. *imphalum*. The PCR assay targeting a 47-kDa gene region was performed by utilizing previously published primers OtsuFP630 (AACTGATTTTATTCAACTAATGCTGCT) and OtsuRP747 (5’- TATGCCTGAGTAAGATACRTGAATRGAATT- 3’) [33]. The amplification was performed in a volume of 25 μL, which contained 2 μL (20 ng) mite DNA as template, 12.5 μL AmpliTaq Gold PCR master mix (catalog no. 4398881; Life Technologies, CA, USA), 1 μl (10μM) each forward and reverse primers and 8.5 μl of nuclease-free water. The amplification program for initial denaturation consisted of 94°C for 10 min, 35 cycles of denaturation at 94°C for 30 s, annealing at 58°C for 30 s, extension at 72°C for 30 s and final extension at 72°C for 10 min. The amplicons were subjected to electrophoresis on ethidium bromide stained 2% agarose gels and visualized with a ChemiDoc-It TS2 imaging system (UVP, Upland, CA). To verify the presence of the pathogen, the amplicons were purified using ExoSAP-IT PCR cleanup reagents and Sanger sequenced at Eton Bioscience, Inc. (Research Triangle Park, NC, USA). DNA sequences were confirmed to be identical to *O*. *tsutsugamushi* by comparison (BLASTn) to *O*. *tsutsugamushi* sequences were 99–100% homologous to nucleotide sequences deposited in GenBank (LS398550) in the NCBI database on August 20, 2018. Each amplification included a negative control (no template DNA) and a positive control, consisting of *O*. *tsutsugamushi* DNA provided by ALR.

### 16S rRNA amplification, library construction, and sequencing

Prior to PCR, DNA was pooled within infection status by life stage (three samples of the same stage/pool), and the samples were subsequently used for 16S rRNA gene amplification of the V3–V4 hypervariable regions. PCR forward and reverse primers were 5′ - TCGTCGGCAGCGTCAGATGTGTATAAGAGACAGCCTACGGGNGGCWGCAG -3′ and 5′ - GTCTCGTGGGCTCGGAGATGTGTATAAGAGACAGGACTACHVGGGTATCTAATCC -3′, respectively [34]. The following number of pooled samples were sequenced: 1) infected larvae (*n* = 6); 2) infected deutonymphs (*n* = 7); 3) infected adult males (*n* = 5); 4) infected adult females (*n* = 8); 5) uninfected larvae (*n* = 7); 6) uninfected deutonymphs (*n* = 6); 7) uninfected males (*n* = 6); 8) uninfected females (*n* = 6), 9) larvae from the infected colony but PCR negative (*n* = 2); 10) males from the infected colony but PCR negative (*n* = 3); and 11) Collembola eggs (*n* = 8) (see S1 File). A 16S rRNA sequencing library was constructed according to Illumina’s 16S rRNA metagenomics sequencing library preparation protocol (Illumina, San Diego, CA, USA). Constructed 16S rRNA metagenomic libraries were quantified with Quant-iT PicoGreen (Molecular Probes, Inc. Eugene, OR, USA), and the libraries were normalized and pooled prior to sequencing. These samples were then sequenced with a 300 paired-end MiSeq run at the Microbiome Core Facility in The School of Medicine, University of North Carolina at Chapel Hill (Chapel Hill, NC, USA).

### Microbiome sequence accession numbers

Raw sequences were submitted to the NCBI read archive under SRA database accession number SRS3073239.

### Bioinformatics data processing

Primary processing of sequencing reads was performed using Quantitative Insights Into Microbial Ecology (QIIME, version 1.9.0) [35, 36]. Paired end V3-V4 sequence reads were joined using fastq-join with the default QIIME settings. Demultiplexing of paired fastq reads was performed using default QIIME parameters, which removed reads with an average Phred quality score less than 20. *De novo* chimera detection and removal were performed using usearch 6.1 [37]. Sequences were trimmed to 318 bp prior to denoising with deblur (a sub-OTU approach), which designated each unique bacterial sequence (100% sequence identity) as a new sub-OTU [38]. Sub-OTUs with <10 reads total in all samples combined were removed. Sequences were matched against the Greengenes 13.8 database using uclust [39, 40]. Sequences were aligned against the Greengenes reference using PyNAST [41] and the alignment was filtered to remove gaps. A phylogenetic tree was created using FastTree 2.1.3 [42]. Rarefaction curves and taxonomy plots were created in QIIME. To ensure an even sampling depth, each sample was rarefied to 12,000 sequences at 30 iterations, and the consensus rarefied OTU table was used for rarefaction analysis to measure α-diversity using the three different indices: 1) the Shannon index, which is based on the abundance and evenness of the observed taxa; 2) number of OTUs, which is a measure of species richness; and 3) the phylogenetic diversity of the samples, which is an unweighted measure of the branch length spanned by a phylogenetic tree of the observed sequences. Taxonomic distributions across sample categories were calculated using the summarize_taxa_through_plots.py script. Jackknifed β-diversity was calculated using UniFrac [43] and principal coordinate analysis (PCoA) plots were visualized using EMPeror [44]. Non-Metric Multidimensional Scaling analysis of microbial communities was performed using Bray-Curtis dissimilarity values using the PAST3 program [45]. Shared and unique OTUs among adults were visualized using a Venn diagram [46]. Finally, the taxonomic assignments of representative sequences of *Orientia* and Amoebophilaceae OTUs were confirmed by searching BLASTn in the NCBI database.

### Statistical analysis

Significant differences in diversity indices between stages were determined with the nonparametric Wilcoxon Rank-Sum Test performed using JMP Pro 12 (SAS Institute Inc., Cary, NC, USA) at *P* < 0.05. The PERMANOVA was applied to test whether within-group distances were significantly different from the between-group distances. Kruskal-Wallis non-parametric tests (corrected for multiple comparisons via Bonferroni and FDR techniques) were used to determine whether the abundance of OTUs differed among groups using the group_significance.py script in QIIME, after filtering the OTU table to remove OTUs present in less than 10 samples in order to focus the results on more abundant OTUs. Pearson product-moment correlation were performed using the relative abundance of OTUs of *O*. *tsutsugamushi* and bacterial sequence classified in the family Amoebophilaceae using JMP Pro 12.

### Attempted PCR Identification of amoeba and endosymbiont in infected female mites

Examination of OTUs revealed that *O*. *tsutsugamushi*-infected female mites contained a large number of reads of a bacterial sequence classified in the family Amoebophilaceae, suggesting that these mites contained a species of amoeba. Accordingly, we performed amoeba-specific PCR amplifications. Previous studies showed that endosymbiontic bacteria were found intracellularly in a free-living amoeba species, *Acanthamoeba* spp. [47]. Specific primers for *Acanthamoeba* spp. and *Naegleria* spp. along with common amoeba-specific and *Amoebophilus*-specific PCR primers were used for PCR amplification of a putative amoeba species in pooled DNA from infected female mites (S1 Table). DNA extracted from two positive controls was included in PCR assays. DNA was extracted from *Amoeba proteus* purchased from Carolina Biological Supply (Greensboro, NC, USA). *Acanthamoeba* spp. DNA was obtained from Dr. Gregory Booton, Department of Molecular Genetics, Ohio State University, Wooster, OH, USA. All PCR amplifications were performed as described in S1 Table. Inability to amplify any DNA fragment from the infected female colony using amoeba-specific PCR primers suggested that it was unlikely that mite samples harbored an amoeba.

### Cloning and sequence analysis of Amoebophilaceae endosymbiont

The short read length of Illumina sequences limited further taxonomic resolution of the Amoebophilaceae bacterium. To improve classification of the Amoebophilaceae OTU, a nearly full length fragment of the 16S rRNA gene of the bacteria was amplified using the universal bacterial primers, 27f and 1492r [48]. DNA from two pooled (ILF01 and ILF02) infected female samples was used. The PCR amplification conditions used were as described by Ponnusamy et al. [49]. Amplified DNA from each reaction was separated in 1.2% agarose gels, stained with ethidium bromide, and photographed using a GelDoc IT2 system (UVP). To generate a 16S clone library, PCR products were cleaned up using AMPure XP beads (Beckman Coulter Genomics, Indianapolis, IN, USA) and purified DNA from amplicons was inserted into the plasmid vector pGEM-T (Cat. No A3610, pGEM–T Vector System II; Promega) as specified by the manufacturer. White colonies were picked and checked for the presence of the insert by amplifying the clones with universal vector primers M13F (CCCAGTCACGACGTTGTAAAACG) and M13R (AGCGATAACAATTTCACACAGG). For each sample, 10 clones with inserts of the expected length were Sanger sequenced at Eton Bioscience, Inc. (Research Triangle Park, NC, USA). Clones with DNA sequences sharing more than 97% identity with GenBank sequences were assigned to that phylotype.

### Phylogenetic analyses of Amoebophilaceae sequences

BLASTn and nucleotide sequence match analysis were used to compare nearly full-length nucleotide sequences of 16S rRNA clones to those in the GenBank database. The 16S rRNA gene sequences obtained were subjected to BLASTn searches (http://blast.ncbi.nlm.nih.gov/) that unambiguously indicated an affiliation with a species of Amoebophilaceae, “*Candidatus* Cardinium hertigii, an endosymbiont of *Acanthamoeba* spp.”, with an identity of 90%. Therefore, an alignment was performed with 12 sequences from the clones, five sequences of other Alphaproteobacteria and “*Candidatus* Sulcia muelleri” sequence as an out group obtained from the NCBI (http://www.ncbi.nlm.nih.gov/) database by BLASTn analysis on November 4, 2017. Multiple alignments were performed by using the clustal_x program, and evolutionary distances were calculated using the Kimura two-parameter model [50]. For reconstructing neighbor-joining and maximum-likelihood phylogenetic trees, the following statistical methods were used. For NJ, the Kimura 2-parameter model, uniform rates and pairwise deletion was used. For ML, the Kimura two-parameter model with uniform rates and the heuristic search algorithm nearest-neighbour-interchange with complete deletion was used. Bootstrap analyses, consisting of 1,000 iterations with the MEGA 6 software package [51], were performed to evaluate the robustness of tree topologies. Sequence data for the 12 clones have been deposited in the NCBI (accession numbers MH093818 to MH093829).

## Results

### Confirmation of *O*. *tsutsugamushi* in infected *L*. *imphalum*

To confirm infection in the mites used in our study, 103 individual mites from the infected colony were screened for *Orientia tsutsugamushi* using a conventional PCR assay. Subsequently, the amplified PCR products were sequenced and confirmed to be from *O*. *tsutsugamushi*. The proportions of infected mites of each life stage were: 75% of larvae (15/20), 95% of deutonymphs (24/25), 53.5% of male adults (15/28), and 90% of female adults (27/30). We then analyzed the microbiome of infected and uninfected larvae, deutonymphs, and male and female *L*. *imphalum* mites by 16S rRNA gene sequencing.

### Overview of diversity in the *L*. *imphalum* bacterial microbiome

Illumina sequencing produced 7,242,598 reads. Following paired-end joining, quality filtering, chimera removal and denoising with deblur, 2,459,696 reads from 65 pooled samples remained. Within these data, sequence reads were clustered into 14,872 sub-operational taxonomic units (sOTUs) and assigned to 815 OTUs of bacterial taxa across all the samples. Overall, the most sequence-abundant at the phylum level were Proteobacteria (43.7%), Bacteroidetes (19.2%), Actinobacteria (18.8%), and Firmicutes (11.6%). In adult mites from the infected colony, we found 4 bacterial phyla with relative mean abundances that were greater than 5%. Proteobacteria (48.0%), Bacteroidetes (28.5%), Actinobacteria (12.7%), and Firmicutes (7.4%) represented 96.6% of the sequences. In comparison with uninfected groups, Proteobacteria (39.1%), Actinobacteria (24.9%), Firmicutes (15.8%) and Bacteroidetes (10%) represented 89.8% of the total number of sequences.

### Bacterial community diversity, richness and evenness of infected and uninfected *L*. *imphalum*

**Fig 1:**
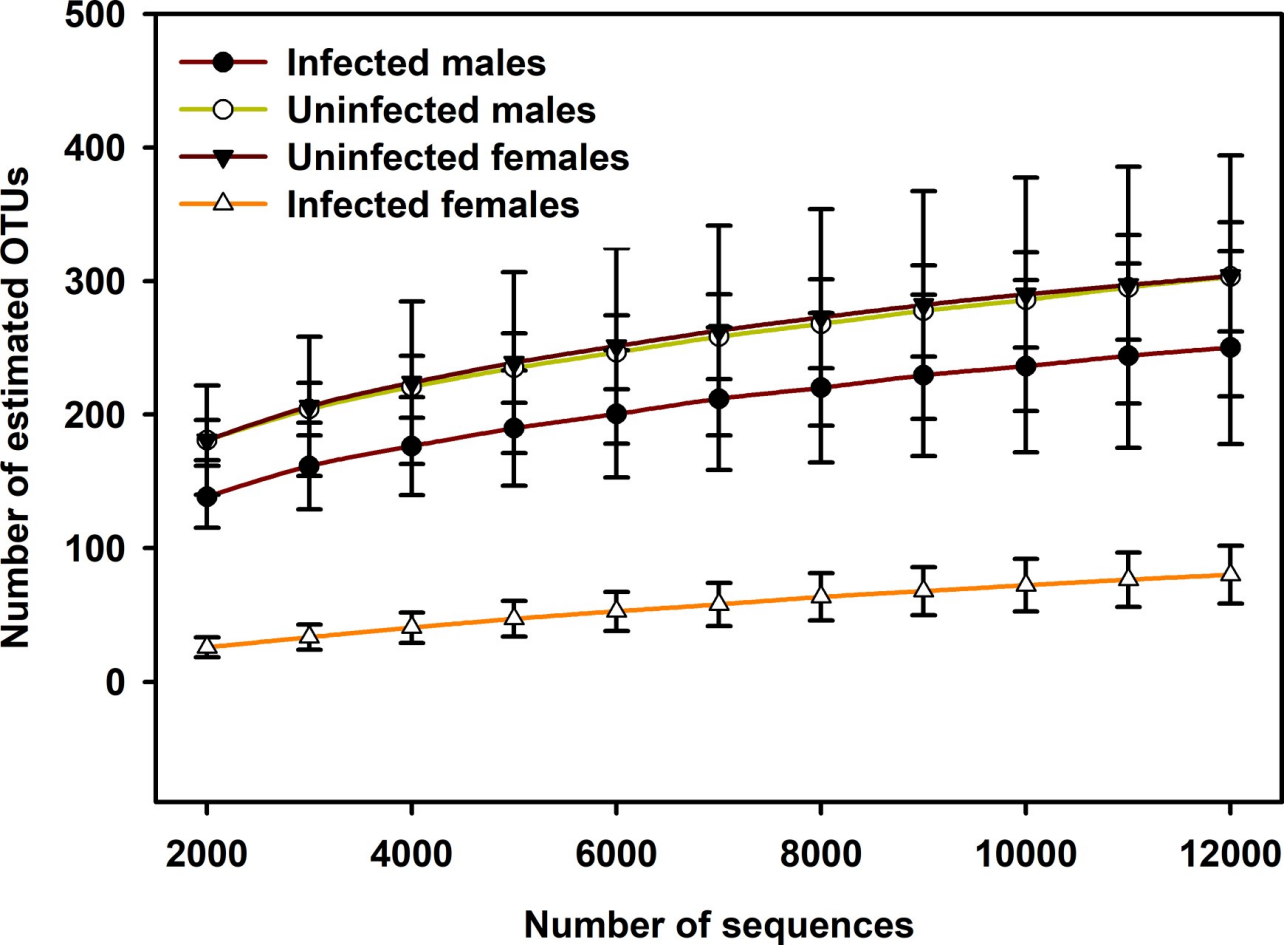
Rarefaction curves of adult mites infected and uninfected with *Orientia tsutsugamushi*. Number of observed OTUs in the 16S rRNA gene sequences of infected and uninfected adults for different rarefaction levels. Error bars are one SE of mean values.

**Fig 2:**
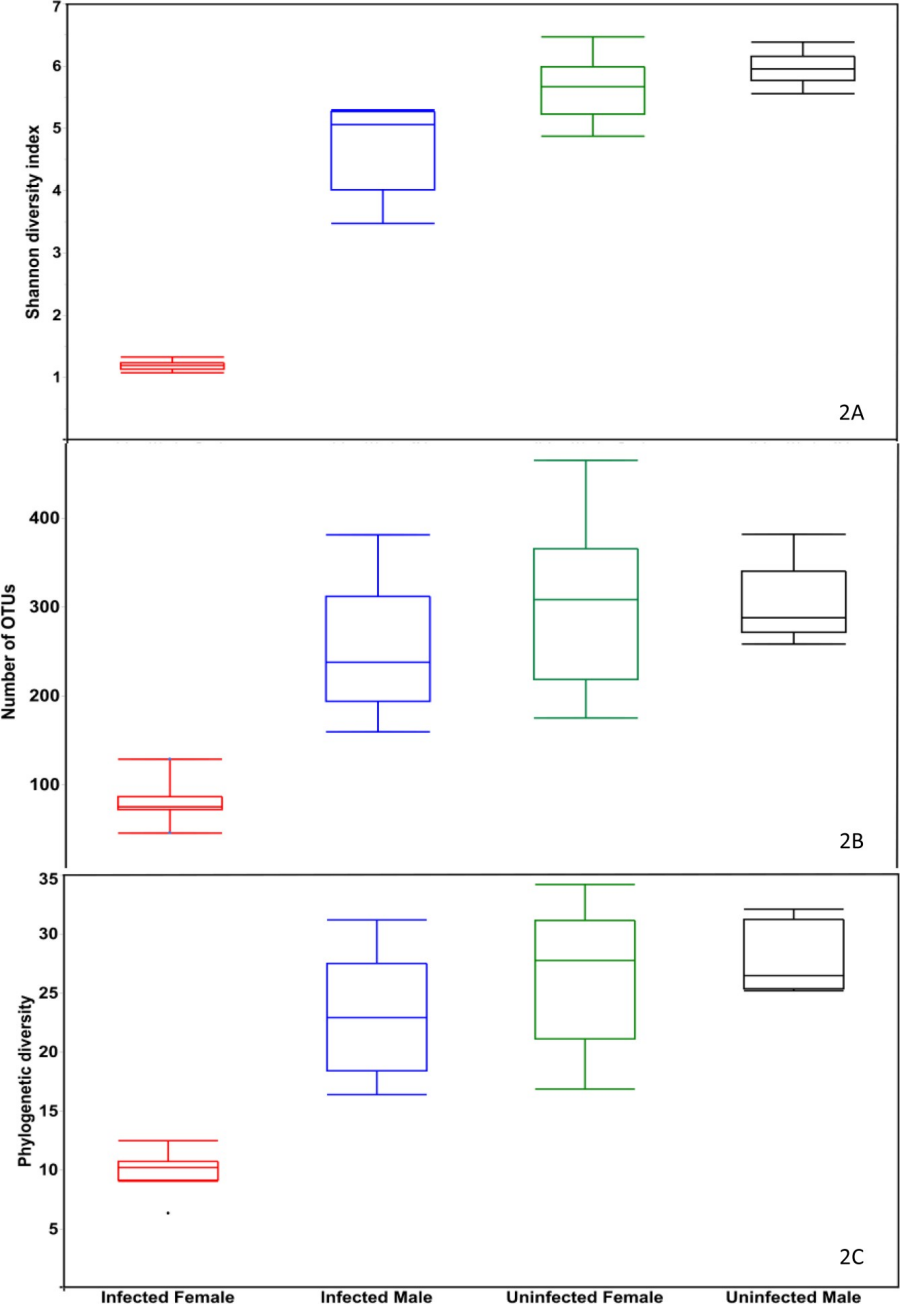
Alpha diversity among adult mites infected and uninfected with *Orientia tsutsugamushi*. Boxplots show the Shannon diversity index (A); the number of observed OTUs (B) and phylogenetic diversity (PD) of the tree (C).

The asymptotic shape of the rarefaction curves suggests that sequencing depth was sufficient to capture the entire bacterial diversity (Fig 1 for all adults, S1 Fig for all stages). Among adults, bacterial community α-diversity analyses revealed significant differences (Wilcoxon Rank-Sum Test, *P* < 0.0008) in Shannon diversity (*H*) indices between infected and uninfected mites (Fig 2A). Specifically, within female adult samples, we observed significantly lower (Wilcoxon Rank-Sum Test, *P* < 0.0024) bacterial diversity in females infected with *O*. *tsutsugamushi* (*H* = 1.19 ± 0.15) than in females not infected with the pathogen (*H* = 5.64 ± 0.18). Additionally, the combined infected female and male samples had significantly lower (Wilcoxon Rank-Sum Test, *P* < 0.0001) species diversity (*H* = 2.55 ± 0.37) than the corresponding uninfected samples (*H* = 5.80 ± 0.39) (Fig 2A for all adult, S2 Fig for Shannon diversity indices of all stages). The number of observed OTUs in infected adults was significantly lower (Wilcoxon Rank-Sum Test, *P* < 0.0008) than in uninfected adults (Fig 2B for all adults, S3 Fig for all stages). Likewise phylogenetic diversity was significantly lower (Wilcoxon Rank-Sum Test, *P* < 0.0002) in the microbiome of infected females compared with uninfected females (Fig 2C for all adults) and across all life stage (Wilcoxon Rank-Sum Test, *P* < 0.05) (S4 Fig).

**Fig 3:**
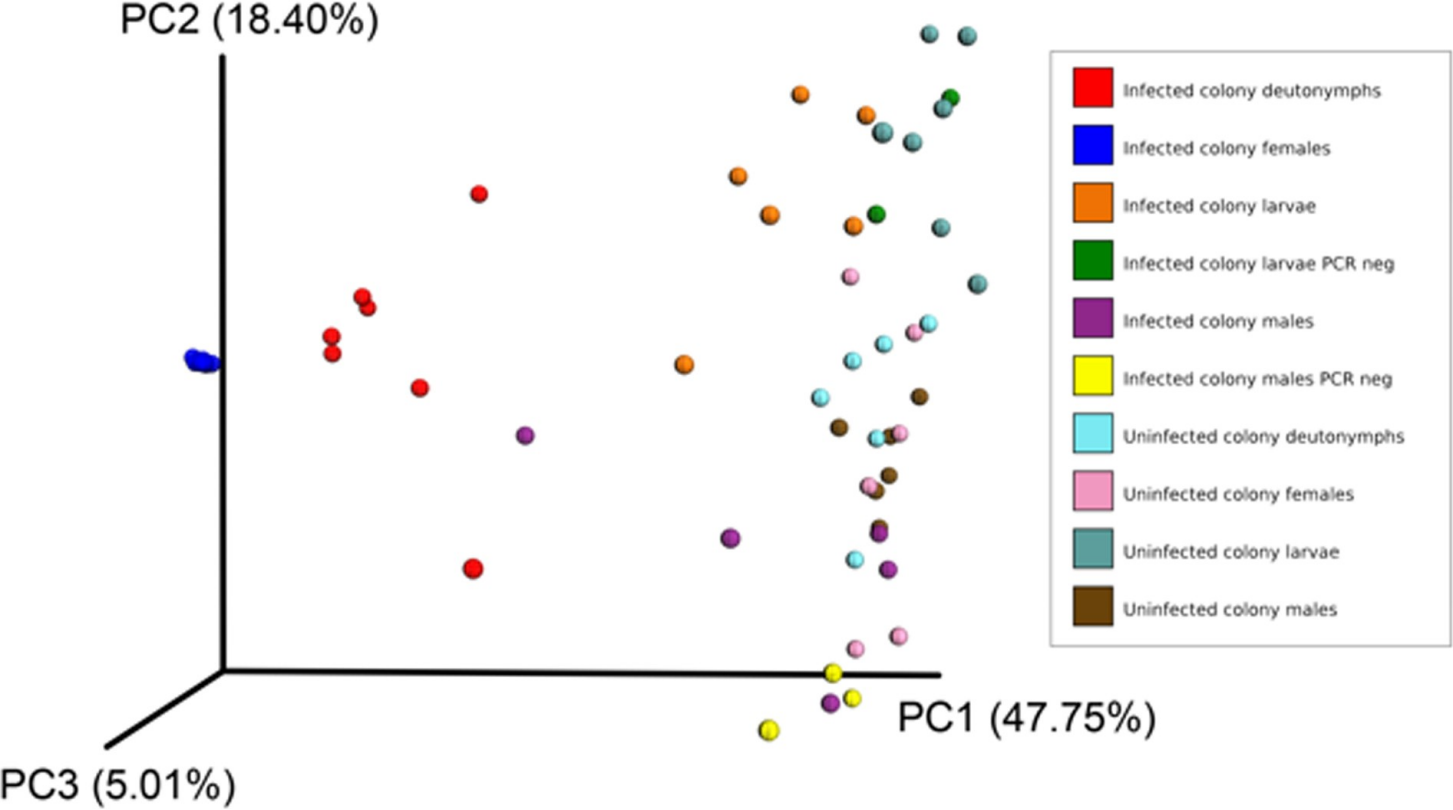
Weighted Principal Coordinates Analysis (PCoA) of the bacterial species observed in different stages of *L*. *imphalum* mites.

To estimate β-diversity between infected and uninfected mites, we used both the unweighted (sensitive to rare taxa) and weighted (based on abundances of taxa) UniFrac distance metric. These analyses revealed that the majority of the variation in bacterial diversity across the samples could be attributed to infection with *O*. *tsutsugamushi* (weighted PCoA, Fig 3; unweighted PCoA, S5 Fig). The uninfected mites were distributed along PC2 at the end of PC1, indicating that their microbiomes had similar community OTU structure. For the infected mites, the larvae and males were loosely distributed in the plane of PC1and PC2. Except for males, as physiological age increased from larvae to deutonymphs, the mites were distributed in a gradient along the plane of PC1 and PC2. However, females were tightly clustered at the center of PC1 at the intersection of PC1 and PC2, indicating that the composition of their microbiome was more homogeneous than for other infected stages (Fig 3). A permutational multivariate analysis of variance found a significant difference (PERMANOVA, *P* = 0.001) between the mites that were grouped by life stage and infection status. Similarly, pairwise comparisons of infected and uninfected females for the OTU composition of the bacterial microbiomes were highly significant (*P* = 0.001). We also separately sequenced mites from the infected colony that were PCR-negative for *O*. *tsutsugamushi*. PCoA analysis did not reveal any significant difference in microbiome OTU community structure between these mites and mites from the uninfected colony.

A supplemental analysis, nonmetric multidimensional scaling using Bray-Curtis dissimilarities, showed that the majority of infected samples clustered together and away from the uninfected colony samples (S6 Fig). The separation of infected and uninfected samples was statistically significant (PERMANOVA, *P* = 0.001). Furthermore, uninfected females from the infected colony clustered together with uninfected samples, indicating that PCR-negative and uninfected colony mites had similar microbiome community structure (S6 Fig). We also tested for significant variation in the frequency of occurrence of individual OTUs between the infected and uninfected adult groups (See S2 File). When all life stages were compared together, 117 OTUs were differentially abundant between groups (Kruskal-Wallis, FDR corrected *P* < 0.05). Post-hoc analyses were performed to determine whether infection with *O*. *tsutsugamushi* was responsible for these differences. When only adult groups were compared (i.e., uninfected males vs. infected males vs. uninfected females vs. infected females), 51 OTUs were differentially abundant. When infected adults were compared to uninfected adults (regardless of sex), 52 OTUs were differentially abundant and the difference between the two groups was statistically significant (Kruskal-Wallis, FDR corrected *P* < 0.05). S7 Fig presents a Venn diagram of unique and shared OTUs in adult mites. The number of OTUs solely found in each group was as follows: infected female (*n* = 181), infected male (*n* = 282), uninfected female (*n* = 370) and uninfected male (*n* = 309) mites. We found 115 OTUs shared by all adult groups. Notably, only four OTUs were uniquely shared between the infected and uninfected female mites and 11 OTUs were uniquely shared between infected and uninfected male mites.

### Microbial community composition of the infected *L*. *imphalum* colony

**Fig 4:**
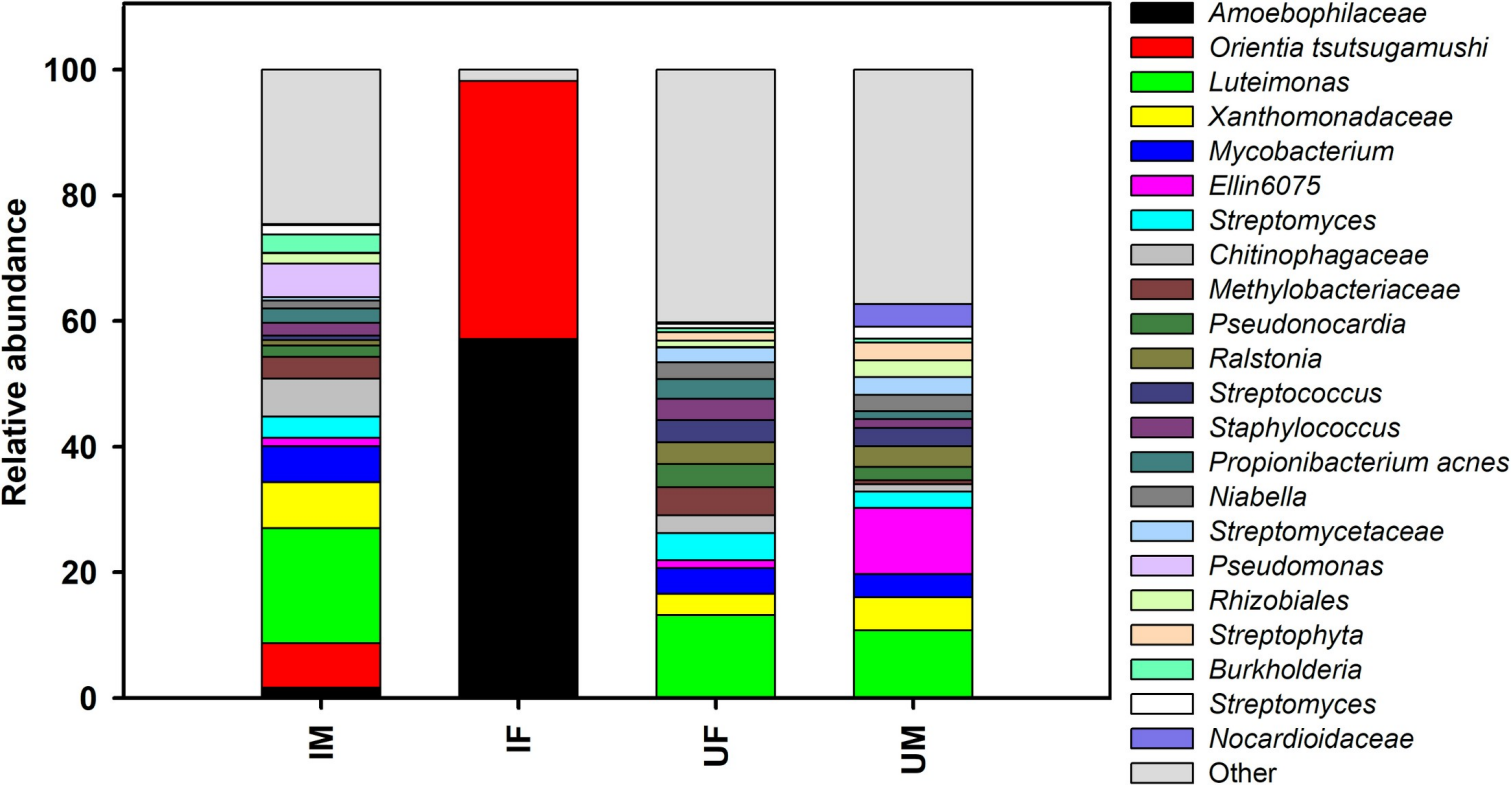
Relative abundance of major bacteria in infected and uninfected male and female mites at the genus level. Bars show proportions of each taxa. ‘Others’ group shows all genera with relative abundance below 1% over the total number of reads. Abbreviations: IF, infected females; IM, infected males; UF, uninfected females; UM, uninfected males.

**Fig 5:**
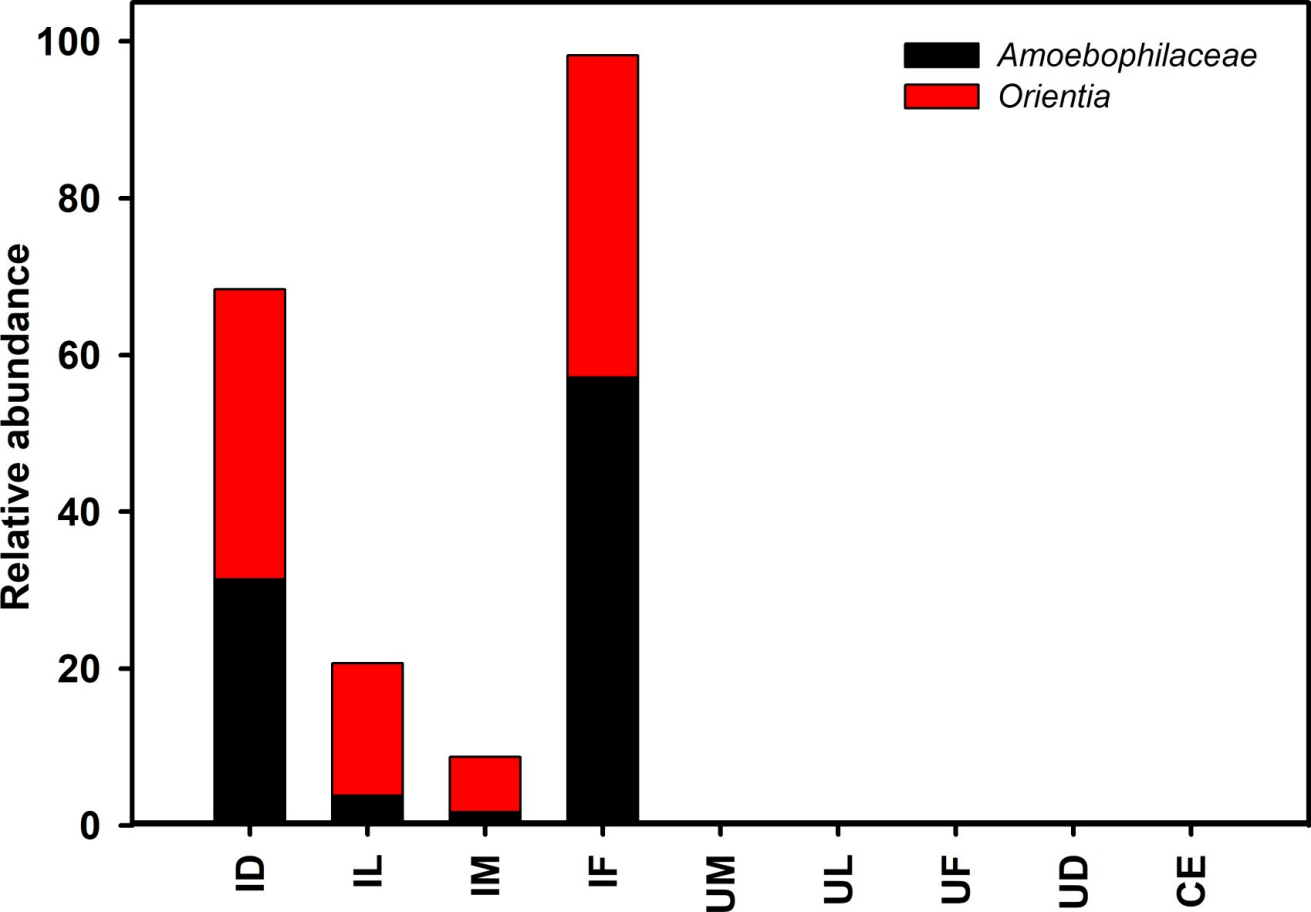
Relative abundance of *Orientia* and Amoebophilaceae OTUs as average across different groups. Abbreviations: ID, infected deutonymphs; IF, infected females; IL, infected larvae; ILPCRN, infected larvae PCR negative; IM, infected males; IMPCRN, infected males PCR negative; UD, uninfected deutonymphs; UF, uninfected females; UL, uninfected larvae, UM, uninfected males, CE, Collembola eggs.

Sequences identifying to *O*. *tsutsugamushi* and to an unidentified genus of the family Amoebophilaceae together made up 98.2% of sequences in infected female mites (Fig 4; S8 Fig; see S3 File for groups; see S4 File for individual pooled samples). For adult male mites from the infected colony, we found 6 bacterial taxa with average relative abundances that were greater than 5%; specifically the family Xanthomonadaceae (genus *Luteimonas* 18.3%), Xanthomonadaceae (7.4%), Rickettsiaceae (genus *Orientia*, 7.1%), Chitinophagaceae (6.1%), Mycobacteriaceae (genus *Mycobacterium*, 5.7%), and Pseudomonadaceae (genus *Pseudomonas*, 5.4%). For the infected deutonymphs, we found only 3 bacterial taxa with a relative abundance greater than 5%: genus *Orientia* (36.94%), family Amoebophilaceae (31.4%) and genus *Stenotrophomonas* (6.8%). For the infected larvae, we found only 3 bacterial taxa with a relative abundance greater than 5%: genus *Orientia* (16.8%), *Ralstonia* (8.8%) and *Propionibacterium* (7%). Notably, there was a significant positive correlation (Person product-moment correlation, *r* = 0.93, *P* < 0.0003) between the presence of the *O*. *tsutsugamushi* pathogen and the Amoebophilaceae bacterium in infected mite samples, suggesting a mutualistic relationship exists between the two bacterial species. BLASTn results for the *Orientia* representative OTU sequences were 100% similar to *O*. *tsutsugamushi* isolate AS/RTN14836/16 (Accession no KY583502.1). *Orientia tsutsugamushi* sequences were most abundant in adult females, followed by deutonymphs, larvae and male mites were found to have the lowest number of reads (Fig 5).

### Microbial community composition of the PCR-negative male and larval mites from the *Orientia* infected colony

Some male mites from the *Orientia* infected colony line were PCR-negative; by 16s RNA gene sequencing, we found very low numbers of sequences of genus *Orientia* (0.01%) and Amoebophilaceae (0.06%) (S3 File). Other notable taxa with a relative abundance greater than 5% included family Weeksellaceae (16.6%), Chitinophagaceae (15.4%), Xanthomonadaceae (9.1%) and Xanthomonadaceae (5.4%). For *Orientia* PCR-negative larvae, no *Orientia* sequences and a low number of sequences for family Amoebophilaceae (0.01%) were detected by Illumina sequencing. Additionally, at an average relative abundance of greater than 5%, we also identified Family Propionibacteriaceae (10.8%), Methylobacteriaceae (9.4%), Oxalobacteraceae (7.3%), Staphylococcaceae (6.9%) and Tissierellaceae (6.5%). Thus, Amoebophilaceae were rare even in the *Orientia-*uninfected larvae and adult male mites from the infected colony.

### Microbial community composition of uninfected mites

At the family level, phylotypes present at a mean relative abundance of greater than 2% across all stages (S8 Fig, S3 File) of uninfected mites were Xanthomonadaceae (genus *Luteimonas*, 8.5%), Oxalobacteraceae (genus *Ralstonia*, 7.9%) Propionibacteriaceae (genus Propionibacterium, 4.3%) Mycobacteriaceae (genus *Mycobacterium*, 4.2%), Streptococcaceae (genus *Streptococcus*, 4%), Ellin6075 (3.0%), Xanthomonadaceae (2.9%), Staphylococcaceae (genus *Staphylococcus*, (2.5%*)* order Streptophyta (2.2%), Corynebacteriaceae (genus *Corynebacterium*, 2.1%) and Streptomycetaceae (genus *Streptomyces*, 2.0%*)*. Except for uninfected larvae, the genus *Luteimonas* was most abundant (between 10.3% to 13.1% of sequences) in all other life stages.

### Analyses of Amoebophilaceae 16S rRNA OTUs and phylogenic analysis of cloned nearly full-length sequence

**Fig 6:**
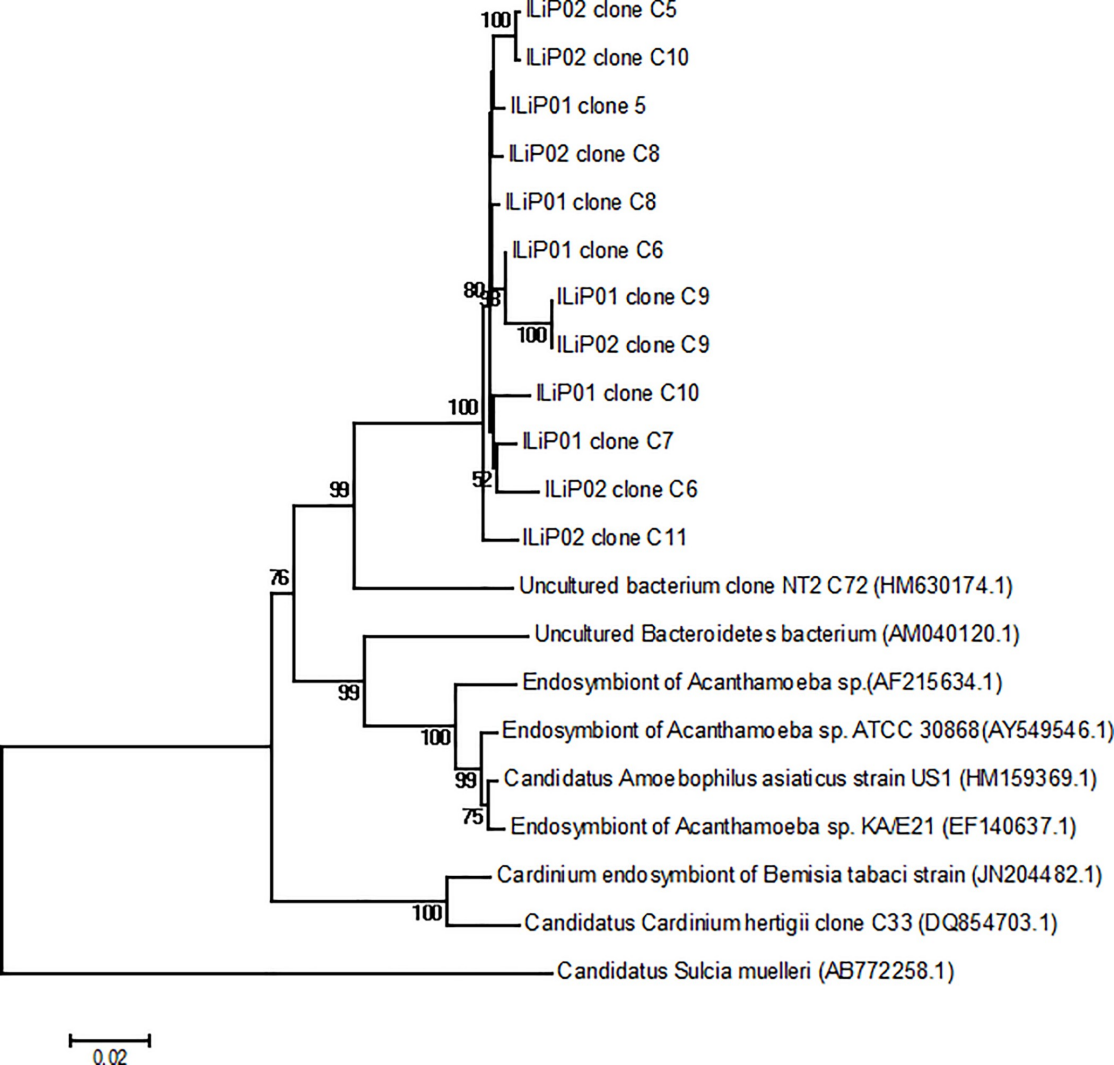
Neighbour-joining tree based on 16S rRNA gene sequences showing the relationship between cloned sequences from infected females, representatives of the genus ‘*Candidatus* Cardinium hertigii’, endosymbiont of *Acanthamoeba* sp and related genera of the family Amoebophilaceae. The sequences were aligned using the Clustal W algorithm. Bootstrap values (>50%), based on 1000 replications, are given at the branching nodes. GenBank accession numbers are shown in parentheses. *Candidatus* Sulcia muelleri (AB772258.1) was used as an outgroup. Bar represents 0.01 substitutions per nucleotide position.

The Amoebophilaceae OTU sequences were compared to sequences in the NCBI database. There were 4 OTUs found among all the samples, but a single OTU representing 99.95% of all Amoebophilaceae sequences reads, was found to be only 93% similar to ‘*Candidatus* Cardinium hertigii’ isolate (Accession no KR026921) and to have 94% identity to uncultured bacterium clone NT2_C72 16S ribosomal RNA gene (Accession number KY517840.1). The cloned nearly full-length 16s rRNA gene sequences from infected female mite adults were comprised of 60% (12/20 clones) Amoebophilaceae and 35% (7/20 clones) *O*. *tsutsugamushi*. These results were similar to the relative proportions of the Illumina sequences. The 12 Amoebophilaceae 16S rRNA gene sequences were composed of approximately 1419 bp each, and the sequences’ maximum nucleotide divergence was 2.5 bp. The highest BLAST matches (BLAST performed on 20^th^ December 2017) for these sequences were 16S rRNA gene sequences from: Endosymbiont of *Acanthamoeba* sp.(AF215634.1) (90.0%), “*Candidatus* Amoebophilus asiaticus” strain US1 (HM159369.1) (90.0%), *Achromobacter aminicus* LMG 26690 T (90.0%), *Cardinium* endosymbiont (HG421081.1) (91.0%), *Cardinium* endosymbiont of *Bemisia tabaci* strain (JN204482.1) (90.6%), “*Candidatus* Cardinium hertigii” clone C33 (91.0%), and uncultured Bacteroidetes bacterium (AM040120.1) (92.0%). The phylogenetic trees based on 16S rRNA gene sequences using neighbour-joining (Fig 6) and maximum-likelihood methods (S9 Fig) showed that the cloned sequences from the infected female samples were clustered in a different clade from the genera “*Candidatus* Amoebophilus spp.” and *Cardinium* endosymbiont in the family Amoebophilaceae. Thus, our phylogenetic analyses showed that the bacterium constitutes a distinct lineage with no closely allied 16S rRNA gene sequences in the DNA databases and clearly represents a novel genus of the family Amoebophilaceae, order Cytophagales, class Cytophagia in the phylum Bacteroidetes. Notably, the phylogenetic patterns generally agreed with the BLASTn search results.

## Discussion

Use of laboratory colonies of *L*. *imphalum* mites made it possible for us to conduct research that would not have been possible with field populations. First, deutonymph and adults mites are free-living and exceedingly difficult to collect in the wild but these life stages are readily available from laboratory colonies. Second, both infected and uninfected colonies were maintained in the laboratory under identical conditions of temperature and relative humidity, and reared on the same diets. All of these variables would likely affect the community structure of the bacterial microbiome of wild populations of mites, and obviously could not be controlled under field conditions.

This is the first study of the global bacterial microbiome of *Leptotrombidium imphalum* mites. We found significant differences in both α- and β-diversity among different developmental stages of mites from *O*. *tsutsugamushi*-infected and uninfected colonies. Specifically, two OTUs identified as *O*. *tsutsugamushi* and a putatively novel genus of the family Amoebophilaceae together made up 98.2% of sequences in infected female mites. Importantly, sequences for these two taxa were markedly low in abundance in PCR-negative male and larval mites from the infected colony line. These study results provide further evidence that a strong causal relationship exists between the Amoebophilaceae symbiont and pathogen infection. Few related studies of mites have used high-throughput sequencing to describe the community structure of the bacterial microbiome. Hubert et al. [52] reported finding 90–99% of sequences in the microbiomes of adults of the ectoparasitic red poultry mite (*Dermanyssus gallinae*), infected with a *Bartonella*-like bacterium, to be comprise of just 10 OTUs. In a related study, [53] found that low bacterial diversity in the house dust mite *Dermatophagoides farinae* was associated with a microbiome that was dominated (99% of sequences) by the endosymbiont *Cardinium*. A less diverse microbiome has also been documented recently in females infected with *Rickettsia* of both *Ixodes scapularis* (blacklegged) tick and *Amblyomma americanum* (lone star tick) [54, 55]. Surprisingly, Collembola eggs (*Sinella curviseta*), which were used as food for the laboratory colonized *L*. *imphalum* mites analyzed in the present study, exhibited higher OTU diversity than both infected and uninfected mites. Even though eggs were cleansed of surface bacteria, the microbial diversity in Collembola eggs was substantially different than in *L*. *imphalum* deutonymphs and adult mites, indicating that bacteria acquired by consuming Collembola eggs do not become established in the mites. Notably in the infected female *L*. *imphalum* mites, sequences identified as *O*. *tsutsugamushi* were most abundant in adult females, followed by deutonymphs and larvae with male adults mites found to have the lowest number of reads. These results are not congruent with those of a recent study [56] of the same laboratory colony showing that the density of *O*. *tsutsugamushi* was highest in larvae compared to other stages. The reasons for the contrasting results are not known but may stem from differences in the methods used to quantify *O*. *tsutsugamushi*. A quantitative real-time PCR assay that targets the 47 kDA antigen gene (htrA) was used by Takhampunya and coworkers to enumerate *O*. *tsutsugamushi* in the mites [56].

A representative sequence from the Amoebophilaceae reads showed 92% similarity to “*Candidatus* Cardinium hertigii”. In general, a minimum identity value for 16S rRNA gene sequences of lower than 94% justifies assignment of the Amoebophilaceae bacterium to a novel genus [57]. *Cardinium* species are endosymbionts of mites and other arthropods [58, 59] and are known to affect host biology by altering fecundity, feminization of infected mites lines, founding parthenogenic lines, and through cytoplasmic incompatibility (see [59] for a review). *Orientia tsutsugamushi* has been classified as a mite endosymbiont [59] because it is transmitted vertically and not acquired horizontally by feeding on infected rodent hosts. As aforementioned, *L*. *imphalum* mites infected with the scrub typhus pathogen have significantly longer development times and decreased fecundity compared to mites that are not infected [29] indicating that the *O*. *tsutsugamushi* has a parasitic relationship with its mite host. *Orientia tsutsugamushi* is often credited with distorting mite sex ratios since many infected colonies, including *L*. *imphalum*, produce almost exclusively female offspring [23, 60]. In one *O*. *tsutsugamushi* infected colony the sex ratio was recovered and males produced again after infected mites were fed an antibiotic [61]. However, some colonies of *Leptotrombidium* infected with *O*. *tsutsugamushi* continue to produce both male and female offspring indicating that *O*. *tsutsugamushi* infection does not always lead to feminization [24]. The discovery of the novel species of Amoebophilaceae and its close association with *O*. *tsutsugamushi* suggests that the feminization of the infected line of *L*. *imphalum* could be caused by the newly discovered species of Amoebophilaceae.

The presence of the Amoebophilaceae and *Orientia* bacteria significantly affected the composition of the microbiome of infected female adult mites. While there are no published studies of bacterial symbionts promoting infection of mite species with pathogenic organisms, non-pathogenic microbial organisms have been reported to facilitate the transmission of pathogens by ticks [19]. A recent study also showed that the pathogen *Anaplasma phagocytophilum* manipulates the microbiota of *Ixodes scapularis* ticks to promote infection [62]. Conversely, it has also been reported that *I*. *scapularis* male ticks infected by a rickettsial endosymbiont had significantly lower rates of infection by *Borrelia burgdorferi* than symbiont-free males, thus showing regulatory interactions among microbial species [63]. In a comparable study [64], the midgut microbiota of *I*. *scapularis* was shown to influence spirochete colonization of ticks. It is worth noting that pathogen enhancement mediated by microbes has also been documented in mosquitoes. Suppression of the midgut bacteria by antibiotic treatment in *Anopheles* mosquitoes reduced O’nyong nyong virus infections [65], indicating that constituents of the microbiota are required for pathogen infection. Subsequent re-infection of live, but not heat-killed bacteria, into antibiotic treated mosquitoes reverted viral titers to levels comparable to untreated controls [65]. A similar pathogen enhancement effect was seen in *Aedes aegypti* mosquitoes re-infected with *Serratia odorifera*, which increased both dengue virus and Chikungunya virus infections [66, 67]. The ability of a variety of bacteria to either enhance or suppress pathogens in insects suggests complex interplay between the host, the microbiome and the pathogen, possibly mediating vector competence.

## Conclusion

Over all life stages, we found that infection of *Leptotrombidium imphalum* mites with *Orientia tsutsugamushi* reduced the abundance and diversity of co-occurring bacterial species, especially in adult female mites. The co-occurrence of *O*. *tsutsugamushi* and a novel species of Amoebophilaceae suggests a mutualistic relationship exists between the two bacterial species. Our results indicate that both bacteria have a profound influence on the bacterial community structure of adult *L*. *imphalum* mites. The occurrence of the Amoebophilaceae bacterium at low levels in uninfected mites suggests the presence of *O*. *tsutsugamushi* is required for the proliferation of the Amoebophilaceae bacterium; however, further research is required to confirm this conclusion. Results of our present study provide a justification for additional research on effects of microbiome community structure on *Leptotrombidium* mite vector competence for *O*. *tsutsugamushi*. The finding that a novel species of Amoebophilaceae was closely associated with *O*. *tsutsugamushi* in *L*. *imphalum* should be confirmed in other mite vectors of scrub typhus.

## Supporting information

S1 Fig Rarefaction curves among different groups of samples. Number of observed OTUs in the 16S rRNA gene sequences of mites for different rarefaction levels. Error bars are one SE of mean values.
(TIF)

S2 Fig Alpha diversity among different groups of samples. **Boxplot show Shannon diversity indices.** Horizontal lines within boxes represent median values. Abbreviations: ID, infected deutonymphs; IF, infected females; IL, infected larvae; ILPCRN, infected larvae PCR negative; IM, infected males; IMPCRN, infected males PCR negative; UD, uninfected deutonymphs; UF, uninfected females; UL, uninfected larvae, UM, uninfected males.
(TIFF)

S3 Fig Alpha diversity among different groups of samples. Boxplot show number of observed OTUs from different groups. Abbreviations: ID, infected deutonymphs; IF, infected females; IL, infected larvae; ILPCRN, infected larvae PCR negative; IM, infected males; IMPCRN, infected males PCR negative; UD, uninfected deutonymphs; UF, uninfected females; UL, uninfected larvae, UM, uninfected males.
(TIF)

S4 Fig Alpha diversity among different groups of samples. Boxplot show phylogenetic diversity indices. Horizontal lines within boxes represent median values. Abbreviations: ID, infected deutonymphs; IF, infected females; IL, infected larvae; ILPCRN, infected larvae PCR negative; IM, infected males; IMPCRN, infected males PCR negative; UD, uninfected deutonymphs; UF, uninfected females; UL, uninfected larvae, UM, uninfected males.
(TIFF)

S5 Fig Unweighted Principal Coordinates Analysis (PCoA) of the bacterial species observed in different stages of mites. (TIF)

S6 Fig Nonmetric multidimensional scaling using Bray-Curtis dissimilarity of OTUs detected in different group of samples. □, infected females; +, infected deutonymphs; ●, infected larvae; ×, infected larvae PCR negative; o, infected males; ◊, infected male PCR negative; Δ, uninfected deutonymphs; ▲, uninfected female; ▯, uninfected larvae; ■, uninfected males.
(TIF)

S7 Fig Venn diagram showing common and unique distribution of 16S rRNA gene OTUs in infected and uninfected male and female *Leptotrombidium imphalum* mites. Abbreviations: IF, infected females; IM, infected males; UF, uninfected females; UM, uninfected males.
(TIF)

S8 Fig Taxonomic composition at the genus level of different group of mite microbiotas. Bars show proportions of taxa per species as average across different group. ‘Others’ group shows all genus level with relative abundance below 1% over the total number of reads. Abbreviations: ID, infected deutonymphs; IL, infected larvae; ILPCRN, infected larvae PCR negative; IM, infected males; IMPCRN, infected males PCR negative; IF, infected females; UD, uninfected deutonymphs; UL, uninfected larvae; UM, uninfected males; UF, uninfected female.
(TIF)

S9 Fig Maximum-likelihood tree based on 16S rRNA gene sequences showing the relationship between cloned sequences from infected female, representatives of the genus Candidatus *Cardinium hertigii*, Endosymbiont of *Acanthamoeba* sp and related genera of the family Amoebophilaceae. The sequences were aligned using the Clustal W algorithm. Bootstrap values (>50%), based on 1000 replications, are given at the branching nodes. GenBank accession numbers are shown in parentheses. *Candidatus Sulcia muelleri* (AB772258.1) was used as a outgroup. Bar, 0.02 substitutions per nucleotide position.
(TIF)

S1 Table List of primers used for attempted identification of amoeba and *Amoebophilus* endosymbionts in infected female mites. (DOCX)

S1 File (XLSX)

S2 File (XLSX)

S3 File (XLSX)

S4 File (XLSX)
