# Supplementary figures and images for: Bacterial microbiome of the chigger mite Leptotrombidium imphalum varies by life stage and infection with the scrub typhus pathogen Orientia tsutsugamushi
Source: PLoS One. 2018 Dec 6;13(12):e0208327. doi: 10.1371/journal.pone.0208327 (PMC6283546; doi:10.1371/journal.pone.0208327)

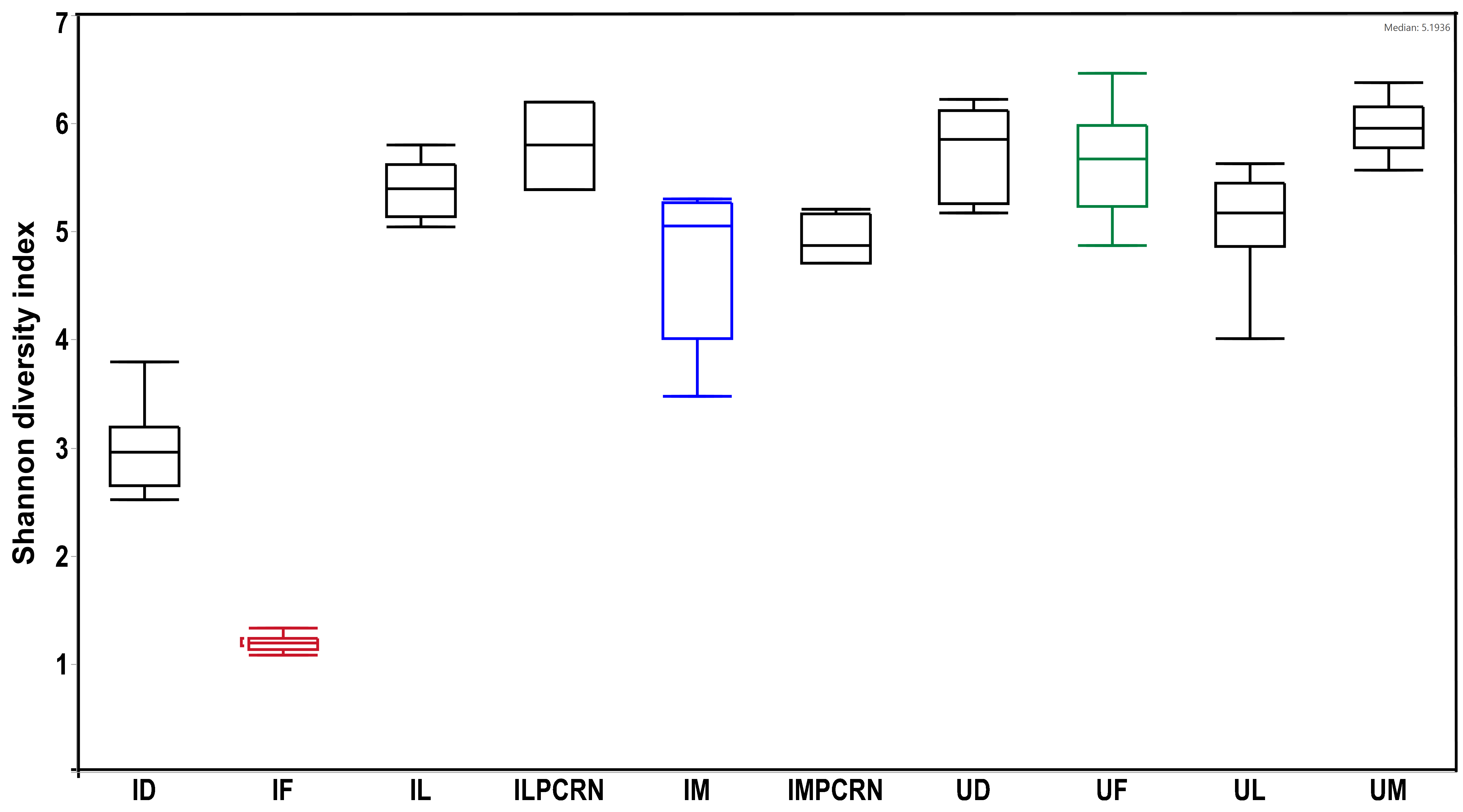

Supplement: S2 Fig — Boxplot show Shannon diversity indices. Horizontal lines within boxes represent median values. Abbreviations: ID, infected deutonymphs; IF, infected females; IL, infected larvae; ILPCRN, infected larvae PCR negative; IM, infected males; IMPCRN, infected males PCR negative; UD, uninfected deutonymphs; UF, uninfected females; UL, uninfected larvae, UM, uninfected males. (TIFF) [file pone.0208327.s002.tiff]

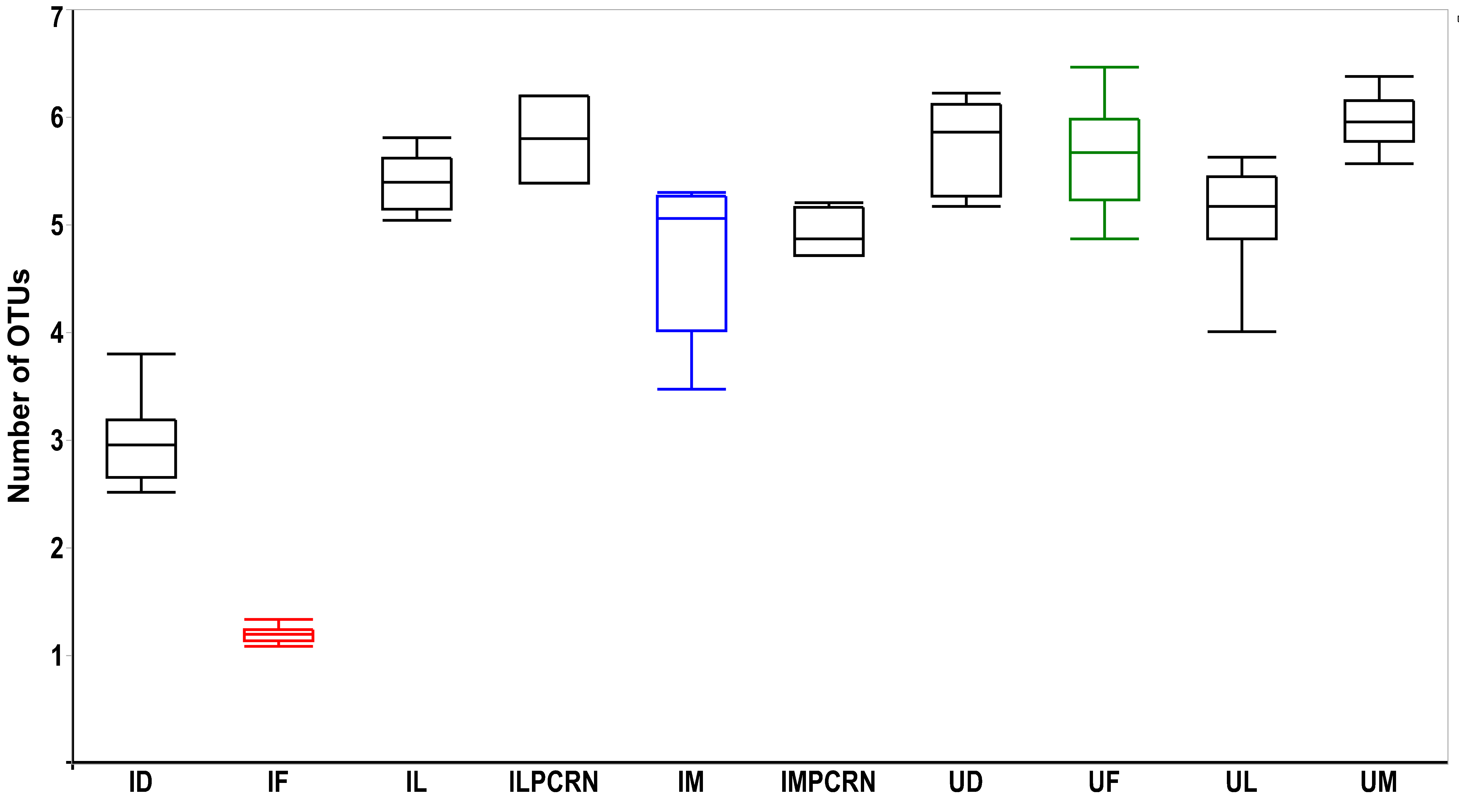

Supplement: S3 Fig — Boxplot show number of observed OTUs from different groups. Abbreviations: ID, infected deutonymphs; IF, infected females; IL, infected larvae; ILPCRN, infected larvae PCR negative; IM, infected males; IMPCRN, infected males PCR negative; UD, uninfected deutonymphs; UF, uninfected females; UL, uninfected larvae, UM, uninfected males. (TIF) [file pone.0208327.s003.tif]

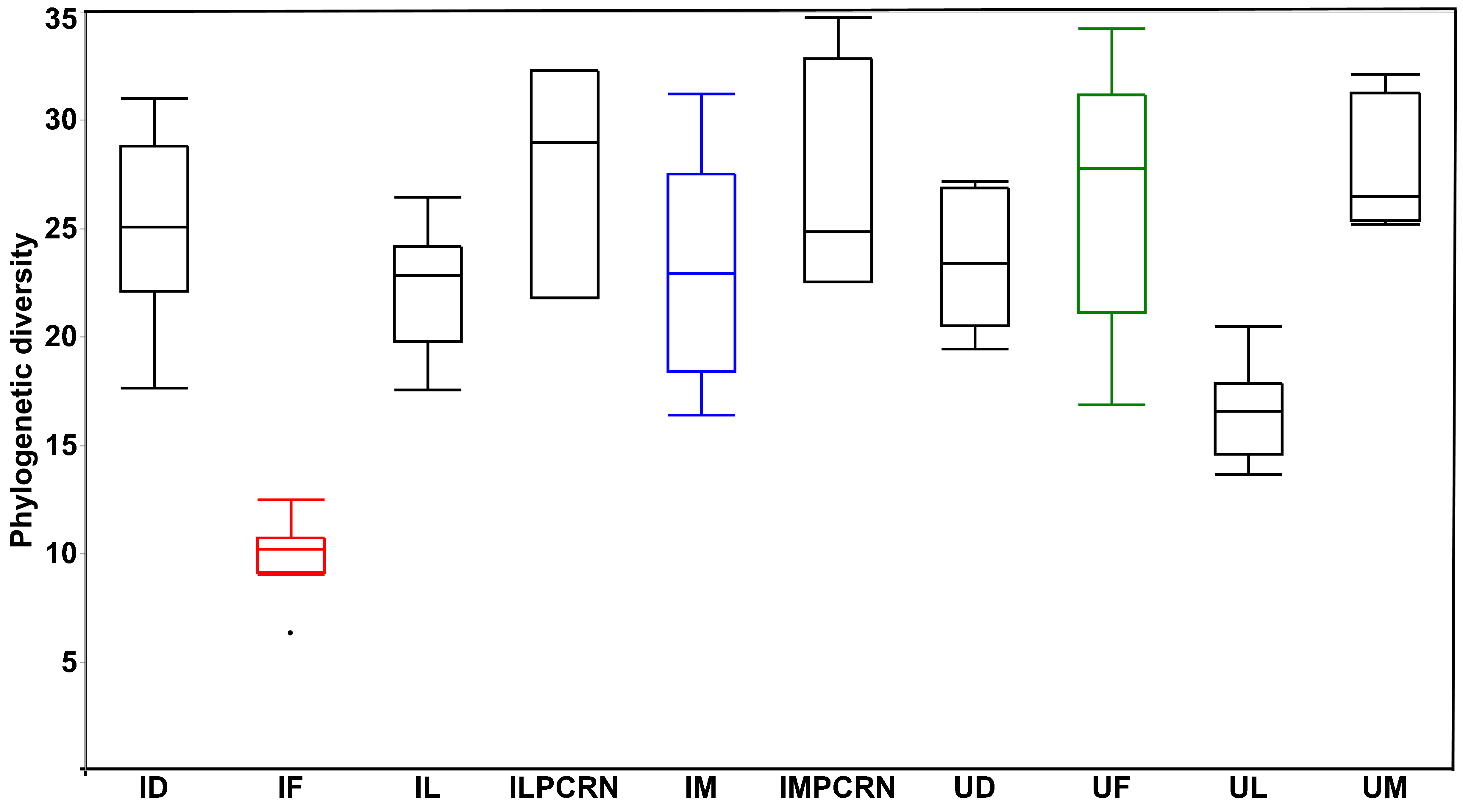

Supplement: S4 Fig — Boxplot show phylogenetic diversity indices. Horizontal lines within boxes represent median values. Abbreviations: ID, infected deutonymphs; IF, infected females; IL, infected larvae; ILPCRN, infected larvae PCR negative; IM, infected males; IMPCRN, infected males PCR negative; UD, uninfected deutonymphs; UF, uninfected females; UL, uninfected larvae, UM, uninfected males. (TIFF) [file pone.0208327.s004.tiff]

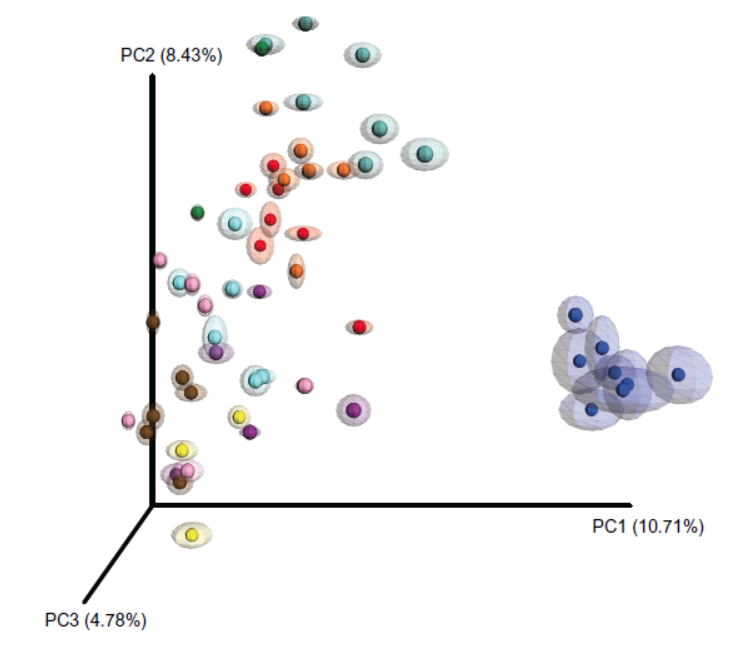

Supplement: S5 Fig — (TIF) [file pone.0208327.s005.tif]

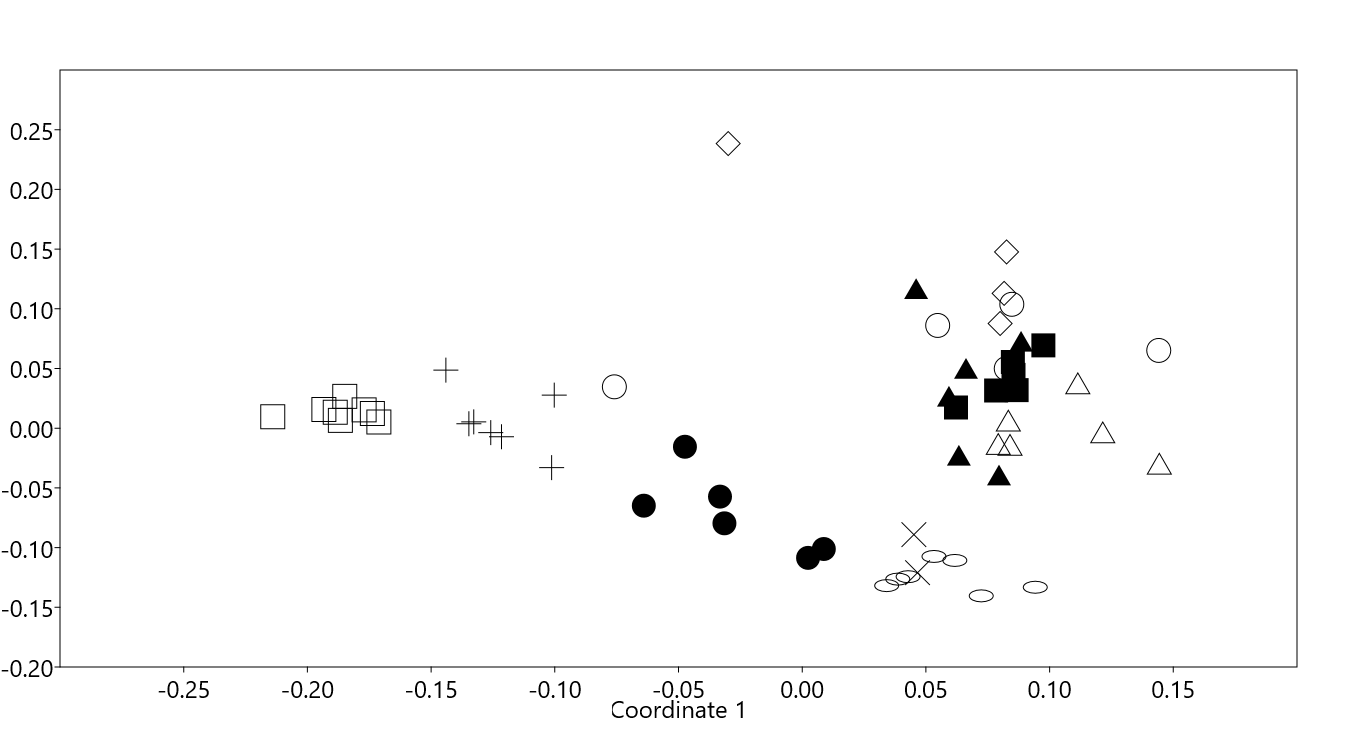

Supplement: S6 Fig — □, infected females; +, infected deutonymphs; ●, infected larvae; ×, infected larvae PCR negative; o, infected males; ◊, infected male PCR negative; Δ, uninfected deutonymphs; ▲, uninfected female; ▯, uninfected larvae; ■, uninfected males. (TIF) [file pone.0208327.s006.tif]

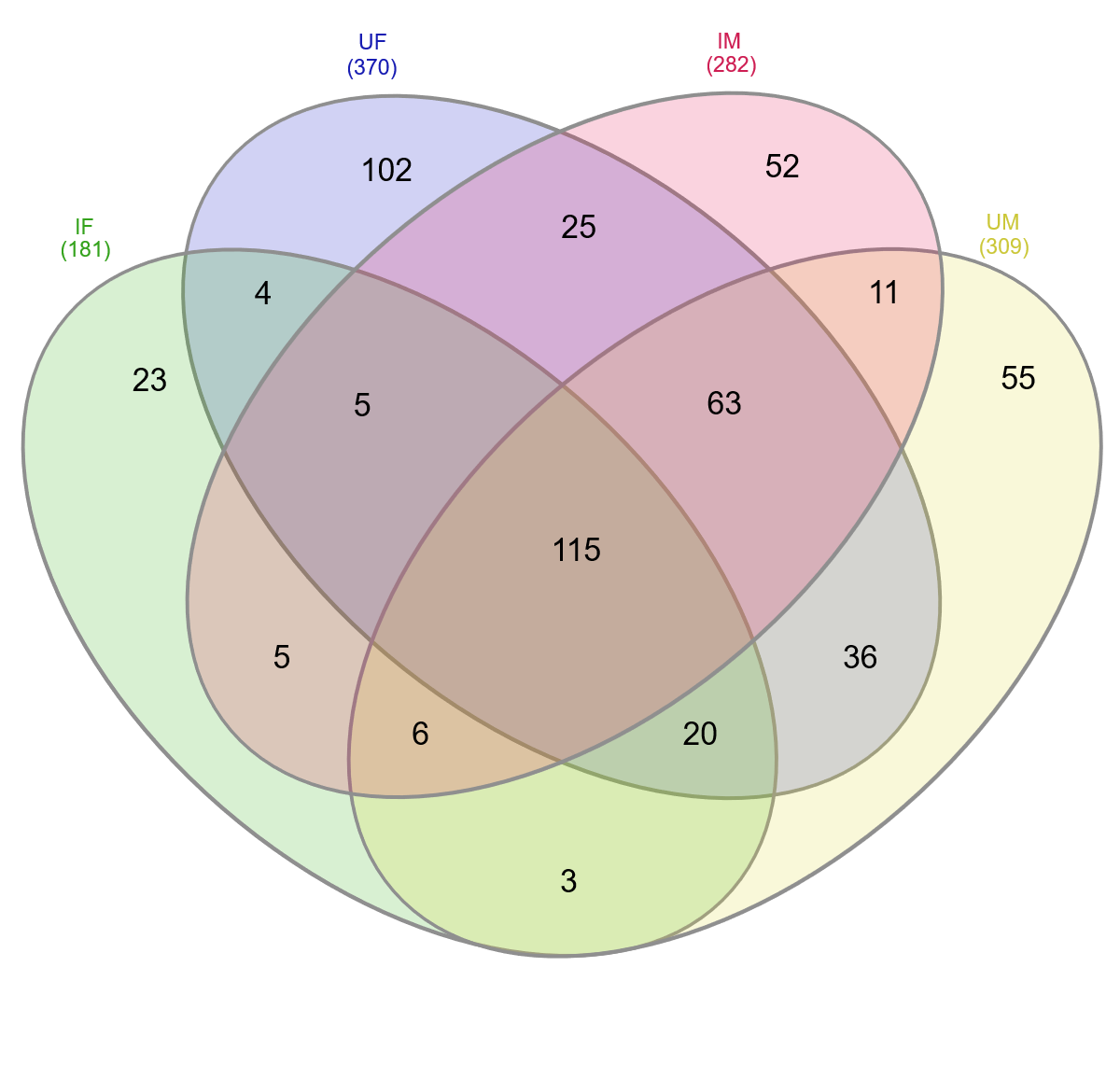

Supplement: S7 Fig — Abbreviations: IF, infected females; IM, infected males; UF, uninfected females; UM, uninfected males. (TIF) [file pone.0208327.s007.tif]

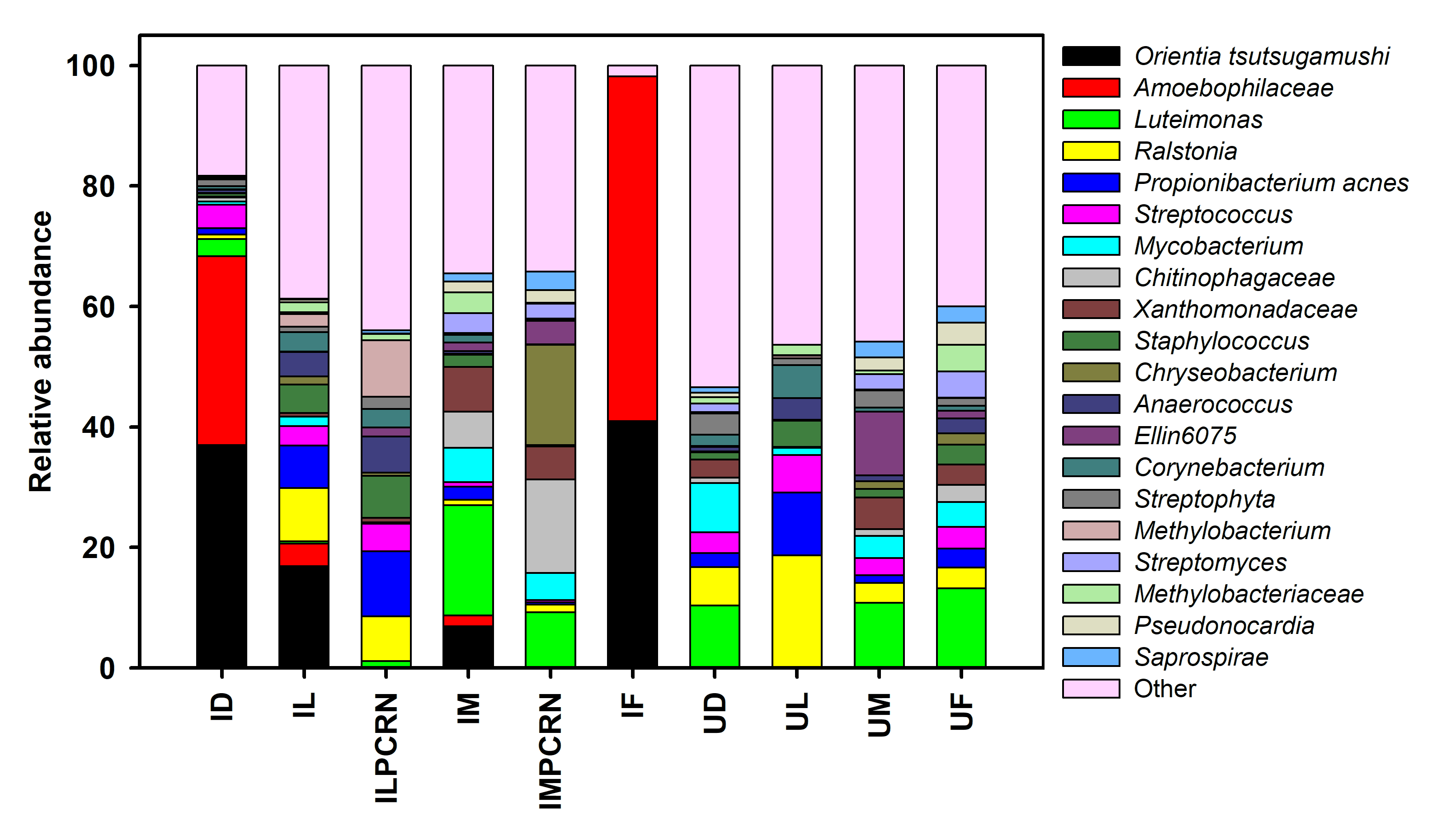

Supplement: S8 Fig — Bars show proportions of taxa per species as average across different group. ‘Others’ group shows all genus level with relative abundance below 1% over the total number of reads. Abbreviations: ID, infected deutonymphs; IL, infected larvae; ILPCRN, infected larvae PCR negative; IM, infected males; IMPCRN, infected males PCR negative; IF, infected females; UD, uninfected deutonymphs; UL, uninfected larvae; UM, uninfected males; UF, uninfected female. (TIF) [file pone.0208327.s008.TIF]

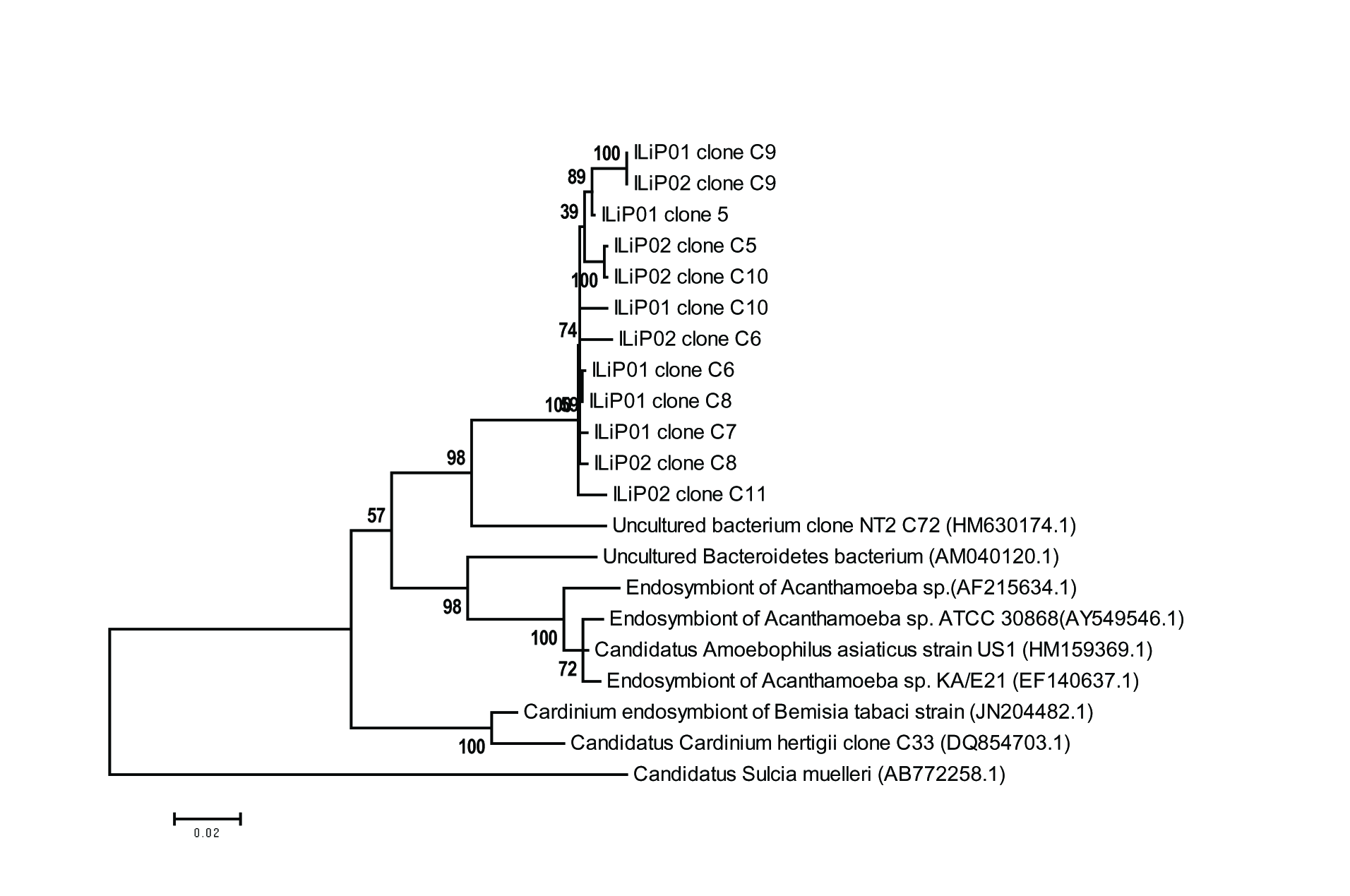

Supplement: S9 Fig — The sequences were aligned using the Clustal W algorithm. Bootstrap values (>50%), based on 1000 replications, are given at the branching nodes. GenBank accession numbers are shown in parentheses. Candidatus Sulcia muelleri (AB772258.1) was used as a outgroup. Bar, 0.02 substitutions per nucleotide position. (TIF) [file pone.0208327.s009.tif]
