# Supplementary material for: Bacterial microbiome of the chigger mite Leptotrombidium imphalum varies by life stage and infection with the scrub typhus pathogen Orientia tsutsugamushi
Source: PLoS One. 2018 Dec 6;13(12):e0208327. doi: 10.1371/journal.pone.0208327 (PMC6283546; doi:10.1371/journal.pone.0208327)
Supplement: S1 Table — (DOCX) [file pone.0208327.s010.docx]

Table S1. List of primers used for attempted identification of amoeba and *Amoebophilus* endosymbionts in infected female mites.

| Specificity | Primer name | Primer sequence (5' –3') | References |
| --- | --- | --- | --- |
| Amoeba | AmeF977 | GATYAGATACCGTCGTAGTC | [[1](#_ENREF_1)] |
|  | AmeR1534 | TCTAAGRGCATCACAGACCTG |  |
| Amoebozoa | 18SF | GTAGTCATATGCTTGTCTC | [[2](#_ENREF_2)] |
|  | 18SR | CGRARACCTTGTTACGAC |  |
| *Acanthamoeba* spp | Ami6F1 | CCAGCTCCAATAGCGTATATT | [[3](#_ENREF_3)] |
|  | Ami6F2 | CCAGCTCCAAGAGTGTATATT |  |
|  | Ami9R | GTTGAGTCGAATTAAGCCGC |  |
| Naegleria spp | NaeF | GAACCTGCGTAGGGATCATTT | [[4](#_ENREF_4)] |
|  | NaeR | TTTCTTTTCCTCCCCTTATTA |  |
| Amoebophilus endosymbiont | AE16SF | GGAACCTTACCTGGGCTAGAATG | In this study |
|  | AE16SF | GTCACTGTCTTCAGGTCCTACCAAC |  |

REFERENCES

1 Liang SY, Ji DR, Hsia KT, Hung CC, Sheng WH, Hsu BM, Chen JS, Wu MH, Lai CH, Ji DD. 2010. Isolation and identification of *Acanthamoeba* species related to amoebic encephalitis and nonpathogenic free‐living amoeba species from the rice field. J Appl Microbiol 109:1422-1429.

2. Schmitz-Esser S, Toenshoff ER, Haider S, Heinz E, Hoenninger VM, Wagner M, Horn M. 2008. Diversity of bacterial endosymbionts of environmental *Acanthamoeba* isolates. Appl Environ Microbiol 74:5822-5831.

3. Thomas V, Herrera-Rimann K, Blanc DS, Greub G. 2006. Biodiversity of amoebae and amoeba-resisting bacteria in a hospital water network. Applied and Environmental Microbiology 72:2428-2438.

4. Pélandakis M, Serre S, Pernin P. 2000. Analysis of the 5.8 S rRNA gene and the internal transcribed spacers in *Naegleria* spp. and in *N. fowleri*. J Eukaryot Microbiol 47:116-121.
